# Comparative Analysis of Clinicopathologic Features of, Treatment in, and Survival of Americans with Lung or Bronchial Cancer

**DOI:** 10.1371/journal.pone.0156617

**Published:** 2016-05-31

**Authors:** Dan Li, Xianglin L. Du, Yinghong Ren, Peijun Liu, Shuting Li, Jiao Yang, Meng Lv, Ling Chen, Xin Wang, Enxiao Li, Jin Yang, Min Yi

**Affiliations:** 1 Department of Medical Oncology The First Affiliated Hospital of Xi’an Jiaotong University, Xi’an, Shaanxi, People’s Republic of China; 2 Department of Respiratory and Critical Care Medicine, The First Affiliated Hospital of Xi’an Jiaotong University, Xi’an, Shaanxi, People’s Republic of China; 3 Department of Epidemiology, Human Genetics and Environmental Sciences, The University of Texas School of Public Health, Houston, Texas, United States of America; 4 Department of Internal Medicine, Shangluo Central Hospital, Shangluo, Shaanxi, China; 5 Department of Translational Medicine, The First Affiliated Hospital of Xi’an Jiaotong University, Xi’an, Shaanxi, People’s Republic of China; 6 Department of Breast Surgical Oncology, The University of Texas MD Anderson Cancer Center, Houston, Texas, United States of America; University of North Carolina School of Medicine, UNITED STATES

## Abstract

Ethnic disparities in lung and bronchial cancer diagnoses and disease-specific survival (DSS) rates in the United States are well known. However, few studies have specifically assessed these differences in Asian subgroups. The primary objectives of the retrospective analysis described herein were to identify any significant differences in clinicopathologic features, treatment, and survival rate between Asian lung cancer patients and lung cancer patients in other broad ethnic groups in the United States and to determine the reasons for these differences among subgroups of Asian patients with lung or bronchial cancer. We searched the Surveillance, Epidemiology, and End Results Program database to identify patients diagnosed with lung or bronchial cancer from 1990 to 2012. Differences in clinicopathologic features, treatment, and DSS rate in four broad ethnic groups and eight Asian subgroups were compared. The study population consisted of 849,088 patients, 5.2% of whom were of Asian descent. Female Asian patients had the lowest lung and bronchial cancer incidence rates, whereas male black patients had the highest rates. Asian patients had the best 5-year DSS rate. In our Asian subgroup analysis, Indian/Pakistani patients had the best 5-year DSS rate, whereas Hawaiian/Pacific Islander patients had the worst 5-year DSS rates. We found the differences in DSS rate among the four broad ethnic groups and eight Asian subgroups when we grouped patients by age and disease stage, as well. Asian patients had better DSS rates than those in the other three broad ethnic groups in almost every age and disease-stage group, especially in older patients and those with advanced-stage disease. In conclusion, we found that clinicopathologic features and treatment of lung and bronchial cancer differ by ethnicity in the United States, and the differences impact survival in each ethnic group.

## Introduction

Worldwide, lung cancer incidence has been decreasing since the 1960s because of a significant reduction in smoking [1], particularly among male individuals. However, it is still the second most common cancer and the leading cause of cancer-related deaths in the United States in female and male individuals, respectively [2].

The lung cancer burden is not shared equally throughout the U.S. population, as ethnic disparities in the disease continue. Investigators have shown that these disparities are reflected in incidence rate, histologic subtype, disease stage, and survival. For example, compared with non-Hispanic white (NHW) patients, black patients have a relatively higher incidence of lung squamous cell carcinoma and adenocarcinoma, resulting in a higher overall lung cancer incidence, especially in younger male black patients [3,4]. Black patients continue to be diagnosed with more advanced lung cancers than are white patients independent of histology, which may contribute to the racial disparities in lung cancer survival in the United States [5,6]. Also, Hispanic white (HW) patients are more likely to be diagnosed with advanced lung cancer and have poorer outcomes than NHW patients [6]. A noteworthy difference in lung cancer incidence and mortality rate is that between Asian Americans, the fastest growing ethnic group in the United States, and other ethnic groups, which some studies have demonstrated. In particular, Asian patients may have lower incidence rates and better survival rates for non-small cell lung cancer (NSCLC) than do non-Asian patients [7–11]. However, few studies have focused on whether the incidence and survival rates differ among the heterogeneous Asian subgroups and NHW, black, and HW patients, especially in large sample sizes over multiyear periods across the United States and for all types of lung cancer, not just in specific areas of the country or for NSCLC.

In a local study in California, Chang *et al* [11] found that Japanese patients with NSCLC (both women and men) had markedly worse overall survival (OS) and disease-specific survival (DSS) rates than did Chinese patients. Also, in a hospital-based study, Finlay *et al* [12] found that foreign-born Chinese and Vietnamese lung cancer patients had more advanced disease stages at presentation, longer durations of pre-diagnosis symptoms, and poorer 2-year survival rates than did non-Asian patients in the Boston, MA, area. In a recent study, researchers examined the differences in cancer-specific mortality of lung cancer among white patients and eight Asian subgroups in the United States and found that most of these subgroups (except Hawaiians) had lower cancer-specific mortality rates than did white patients [13]. However, this study only looked at the cancer-specific mortality among white and Asian American subgroups. Differences in lung cancer incidence and survival among ethnic groups may be attributed to complex interaction of genetic and lifestyle factors [8,14]. Nevertheless, some researchers have presented different hypotheses about such disparities, finding no ethnic differences in histologic subtype, stage, or survival of lung cancer [15,16].

The primary objectives of the retrospective analysis described herein were to identify any significant differences in clinicopathologic features, treatment, and survival rate between Asian lung cancer patients and lung cancer patients in other broad ethnic groups in the United States and to determine the reasons for these differences among subgroups of Asian patients with lung or bronchial cancer.

## Materials and Methods

### Patient selection and data collection

The patient data used in this study were obtained from all 18 U.S. cancer registries in the Surveillance, Epidemiology, and End Results (SEER) Program database (National Cancer Institute) using the SEER*Stat software program (version 8.2.1; http://seer.cancer.gov/seerstat [accessed June 8, 2015]) under a data user agreement. Patient records/information was anonymized and de-identified prior to analysis. The First Affiliated Hospital of Xian Jiaotong University ethics committee review board approved this retrospective study. The SEER database was searched to identify patients whose primary tumor sites were coded as C34.0 or C34.9 (lung and bronchial cancer, respectively) Correct and whose cancers were diagnosed from 1990 to 2012. The SEER 18 registries routinely collect data on patient demographics, primary tumor site, tumor morphology, cancer stage at diagnosis, first course of treatment, and follow-up vital status. Tumor grade was not included in our analysis because it was unknown for more than 50% of the patients. The geographic scope of the SEER database was described previously [17–19]. Five thousand two patients with lymphoma or sarcoma were excluded, as were 5298 patients with race coded as American Indian/Alaska Native or unknown. The age-adjusted SEER incidence rates for lung and bronchial cancer by race and sex from 1992 to 2012 were calculated using the Fast Stats page of the SEER website (http://seer.cancer.gov/faststats/selections.php?series=race,sex; accessed June 11, 2015).

### Statistical analysis

Our primary interest was lung and bronchial cancer patient ethnicity. In the SEER database, ethnicity is categorized into four broad groups: NHW, black, HW, and Asian (including Pacific Islander). In our analysis, Asian patients were further categorized into eight subgroups: Filipino, Chinese, Japanese, Indian/Pakistani, Korean, Vietnamese, Hawaiian/Pacific Islander (Hawaiian, Samoan, Pacific Islander, Tongan, Fijian, Guamanian, Micronesian, Polynesian, Melanesian, Chamorro, Tahitian, Kampuchean, and New Guinean), and other (undefined). The chi-square test was used to assess differences in patient characteristics, management, and outcomes among the four broad ethnic groups and eight Asian subgroups.

The primary endpoint of this study was DSS, which was the number of years from the date of lung cancer diagnosis to the date of cancer-related death, date last known to be alive, or December 31, 2012, whichever came first. OS was the number of years from the date of lung cancer diagnosis to the date of cancer-related death, date last known to be alive, or November 30, 2012, whichever came first. OS and DSS curves for the study patients were calculated using the Kaplan-Meier method. Patients who died during follow-up or survived beyond November 30, 2012, were censored. Multivariable Cox proportional hazards models were used to determine the influence of patient, tumor, and treatment factors of known or potential prognostic value (age at diagnosis, sex, year of diagnosis, ethnicity, tumor stage, tumor grade, primary tumor site, and primary surgery) on DSS. The Stata/SE software program (version 12; StataCorp, College Station, TX, USA) was used for statistical analyses. All tests were two-tailed, and statistical significance was set at p<0.05.

## Results

### Patient and tumor characteristics

We analyzed data on 849,088 patients diagnosed with lung or bronchial cancer in this study. Specifically, we examined data on 672,863 (79.2%) NHW, 91,654 (10.8%) black, 44,526 (5.2%) Asian, and 40,045 (4.7%) HW patients.

The clinicopathologic characteristics of the patients in the four broad ethnic groups are shown in Table 1. We observed marked differences in patient percentages according to cancer stage, age at diagnosis, marital status, sex, use of surgery, location of disease, tumor histology, and use of radiation treatment among the groups. NHW and Asian patients were markedly older at diagnosis than black patients (median age, 70 years versus 66 years). Also, NHW and Asian patients had stage III/IV tumors (75.0% and 78.3%, respectively) less often than did black (81.1%) and HW (79.7%) patients. The proportion of individuals with regional or distant disease was highest among black patients (83.2%). In addition, black patients underwent surgical treatment (17.1%) less often than NHW (22.7%) and Asian (21.3%) patients did. Radiation treatment was more common in black patients (41.7%) than in the other three groups. Adenocarcinoma was the most common histologic type of lung cancer (30.9%) in the entire cohort, and the percentage of this cancer was highest in Asian patients (41.4%). Furthermore, Asian patients had the highest proportion of bronchioalveolar adenocarcinoma (BAC; 4.6%) and the lowest proportions of small cell lung cancer (17.7%), squamous cell lung cancer (15.7%), and large cell lung cancer (3.6%). In comparison, black patients had the highest percentages of squamous cell lung cancer (22.5%) and large cell lung cancer (5.3%), but a lower percentage of adenocarcinoma (30.8%) than in the other three groups and the lowest percentage of BAC (2.2%). In addition, HW patients more often had adenocarcinoma and BAC (33.3% and 3.5%, respectively) than did NHW patients (30.0% and 2.9%, respectively).

**Table 1:** Baseline demographic and clinicopathologic characteristics of the 849,088 study patients.

Characteristic	All patients % (n = 849,088)	NHW % (n = 672,863)	Black % (n = 91,654)	HW % (n = 40,045)	Asian % (n = 44,526)	P
**Age at diagnosis, years**						0.0001^a^
<Mean (median)	69.0 (70.0)	69.7 (71.0)	65.7 (66.0)	68.3 (70.0)	69.0 (70.0)	
**Age group, years**						<0.0001
<30	0.1	0.1	0.1	0.5	0.2	
30–39	0.6	0.5	0.9	1.4	1.2	
40–49	4.4	3.8	7.5	5.6	5.2	
50–59	14.6	13.7	21.7	14.8	14.6	
60–69	28.4	28.2	31.1	27.5	26.4	
70–79	33.1	34.0	26.8	32.1	32.7	
≥80	18.8	19.7	11.9	18.1	19.7	
**Sex**						<0.0001
Female	45.4	46.4	41.0	44.6	40.5	
Male	54.6	53.6	59.0	55.4	59.5	
**Marital status**						<0.0001 ^b^
Single	11.1	9.5	26.2	14.5	8.9	
Married	51.6	55.0	37.2	53.8	66.5	
Other	33.5	35.5	36.6	31.7	24.6	
Unknown	3.8	--	--	--	--	
**TNM stage**						<0.0001 ^b^
I	16.4	20.9	16.3	16.9	18.1	
II	3.3	4.1	3.6	3.4	3.7	
III	22.7	27.5	29.8	26.8	28.0	
IV	39.5	47.5	50.3	52.9	50.2	
Unknown	18.1	--	--	--	--	
**SEER summary stage**						<0.0001 ^b^
Localized	18.3	20.8	16.8	17.3	17.1	
Regional	23.8	26.4	25.8	23.6	23.6	
Distant	49.3	52.8	57.4	59.1	59.3	
Unknown	8.6	--	--	--	--	
**Cancer-directed surgery**						<0.0001 ^b^
Not performed	78.1	77.3	82.9	80.6	78.7	
Performed	21.8	22.7	17.1	19.4	21.3	
Unknown	0.1	--	--	--	--	
**Reason for no surgery**						<0.0001 ^b^
Died	3.7	4.5	3.5	3.5	2.6	
Not recommended	81.7	92.7	93.7	94.2	94.2	
Patient refused	2.5	2.8	2.8	2.3	3.2	
Unknown	12.1	--	--	--	--	
**Radiation treatment**						<0.0001 ^b^
Not received	59.3	62.0	58.3	66.0	62.8	
Received	36.7	38.0	41.7	34.0	37.2	
Unknown	4.0	--	--	--	--	
**Lymph node status**						<0.0001
Negative	55.9	56.8	50.0	55.1	53.0	
Positive	44.1	43.2	50.0	44.9	47.0	
**Tumor location**						<0.0001
Main bronchus	5.0	5.1	5.1	5.4	4.0	
Upper lobe, lung	47.3	47.1	51.0	43.2	45.7	
Middle lobe, lung	4.1	4.1	4.3	4.0	4.9	
Lower lobe, lung	23.1	23.3	19.8	23.6	25.4	
Overlapping	1.4	1.4	1.5	1.5	1.3	
Lung, NOS	19.1	19.0	18.3	22.3	18.7	
**Tumor histology**						<0.0001
Small cell	22.5	23.2	20.9	21.1	17.7	
Squamous cell	19.7	19.7	22.5	17.3	15.7	
Large cell	4.4	4.3	5.3	3.9	3.6	
Adenocarcinoma	30.9	30.0	30.8	33.3	41.4	
BAC	2.9	2.9	2.2	3.5	4.6	
Other	19.6	19.9	18.3	20.9	17.0	

NOS: not otherwise specified

^**a**^ Kruskal-Wallis equality-of-populations rank test.

^**b**^ calculated after exclusion of patients in the Unknown category.

Of the Asian patients, 24.0% were Filipino, 16.5% were Japanese, 23.7% were Chinese, 10.4% were Hawaiian/Pacific Islander, 7.0% were Korean, 3.2% were Indian/Pakistani, 8.4% were Vietnamese, and 6.8% were other. Table 2 lists the clinicopathologic characteristics of the Asian patients in the eight subgroups. Hawaiian/Pacific Islander patients were the youngest at diagnosis (median age, 66 years), whereas Japanese patients were the oldest (median age, 74 years). We observed that lung cancer was diagnosed in 67.2% of Japanese patients older than 70 years but only 48.3% of Hawaiian/Pacific Islander patients in that age group (p<0.0001). Moreover, Japanese patients had the highest proportions of stage I/II disease (23.8%) and localized disease (18.2%) followed by Indian/Pakistani patients (23.6% for stage I/II disease and 17.7% for localized disease). The proportions of stage III/IV disease and regional/distant disease were highest in Hawaiian/Pacific Islander patients (80.1% and 84.2%, respectively). Additionally, Indian/Pakistani patients had the highest rate of surgical treatment (24.8%) followed by Japanese patients (22.9%), whereas Hawaiian/Pacific Islander patients had the lowest rate of surgical treatment (18.4%) but highest rate of radiation treatment (43.4%). Chinese, Filipino, Indian/Pakistani, and Vietnamese patients were more likely to be diagnosed with adenocarcinoma and BAC and less likely to be diagnosed with small cell lung cancer and squamous cell lung cancer than were Japanese, Korean, and Hawaiian/Pacific Islander patients.

**Table 2:** Patient, tumor, and treatment characteristics in the Asian subgroups with lung or bronchial cancer.

Characteristic	Filipino % (n = 10,675)	Japanese % (n = 7346)	Chinese % (n = 10,561)	Hawaiian/Pacific Islander % (n = 4639)	Korean % (n = 3108)	Indian/Pakistani % (n = 1434)	Vietnamese % (n = 3759)	Other % (n = 3004)	P
**Age at diagnosis, years**									0.0001^a^
Mean (median)	68.4 (69.0)	72.9 (74.0)	70.6 (72.0)	65.6 (66.0)	68.9 (70.0)	65.2 (67.0)	65.6 (67.0)	67.3 (69.0)	
**Age group, years**									<0.0001
<30	0.2	0.04	0.2	0.2	0.2	1.1	0.3	0.5	
30–39	1.0	0.4	1.2	1.1	0.8	3.2	2.6	1.7	
40–49	4.6	2.0	4.8	7.3	5.0	7.3	8.6	7.2	
50–59	15.8	8.2	12.3	20.7	14.5	17.6	19.8	18.1	
60–69	29.6	22.2	22.7	32.4	28.5	29.8	26.9	24.3	
70–79	32.0	39.6	33.7	27.6	32.1	29.2	27.6	30.7	
≥80	16.8	27.6	25.1	10.7	18.9	11.8	14.2	17.5	
**Sex**									<0.0001
Female	36.6	44.0	42.0	42.4	42.6	33.5	34.4	46.3	
Male	63.4	56.0	58.0	57.6	57.4	66.5	65.6	53.7	
**Marital status**									<0.0001^b^
Single	7.1	10.1	7.3	12.0	8.2	6.8	11.4	11.8	
Married	70.1	60.0	70.3	54.4	68.7	74.7	69.7	64.2	
Other	22.8	29.9	22.4	33.6	23.1	18.5	18.9	24.0	
**TNM stage**									<0.0001^b^
I	17.5	19.5	17.9	16.4	18.1	20.4	17.8	19.0	
II	3.8	4.3	3.5	3.5	4.0	3.2	3.4	3.3	
III	27.8	29.5	27.7	30.3	29.9	25.1	25.6	26.3	
IV	50.9	46.7	50.9	49.8	48.0	51.3	53.2	51.4	
**SEER summary stage**									<0.0001^b^
Localized	16.9	18.2	17.0	15.7	17.1	17.7	17.0	17.9	
Regional	22.9	26.1	22.4	25.4	24.2	25.1	21.3	22.7	
Distant	60.2	55.7	60.6	58.9	58.7	57.2	61.7	59.4	
**Cancer-directed surgery**									<0.0001^b^
Not performed	79.9	77.1	78.5	81.6	77.9	75.2	78.6	78.0	
Performed	20.1	22.9	21.5	18.4	22.1	24.8	21.4	22.0	
**Reason for no surgery**									<0.0001^b^
Died	2.0	3.5	2.4	3.7	2.7	3.1	1.5	1.9	
Not recommended	94.8	93.3	94.3	92.0	94.1	94.1	95.7	95.4	
Patient refused	3.2	3.2	3.3	4.3	3.2	2.8	2.8	2.7	
**Radiation treatment**									<0.0001^b^
Not received	62.3	60.6	64.8	56.6	64.3	62.9	64.4	68.4	
Received	37.7	39.4	35.2	43.4	35.7	37.1	35.6	31.6	
**Lymph node status**									<0.0001
Negative	49.5	55.9	54.8	49.8	50.7	55.5	52.4	57.0	
Positive	50.5	44.1	45.2	50.2	49.3	44.5	47.6	43.0	
**Tumor location**									<0.0001
Main bronchus	4.0	3.6	3.8	4.2	4.9	3.8	3.5	4.7	
Upper lobe, lung	46.6	46.5	45.1	48.1	41.3	44.7	45.0	45.3	
Middle lobe, lung	4.8	4.5	5.4	4.5	4.6	4.6	5.4	4.9	
Lower lobe, lung	24.4	25.3	26.2	22.1	27.4	27.9	27.4	24.9	
Overlapping	1.6	1.5	1.1	1.3	1.2	1.1	1.5	1.2	
Lung, NOS	18.6	18.6	18.4	19.8	20.6	17.9	17.2	19.0	
**Tumor histology**									<0.0001
Small cell	17.1	19.1	15.1	21.9	19.4	18.1	17.4	18.2	
Squamous cell	15.7	18.7	12.4	19.8	20.1	16.0	12.4	12.7	
Large cell	3.4	3.9	4.2	3.5	3.1	3.2	3.4	3.7	
Adenocarcinoma	43.5	35.1	44.5	33.7	36.8	42.0	48.4	44.8	
BAC	4.9	4.2	5.5	2.7	4.3	4.5	5.0	4.5	
Others	15.4	19.0	18.3	18.4	16.3	16.2	13.4	16.1	

NOS: not otherwise specified

^**a**^ Kruskal-Wallis equality-of-populations rank test.

^**b**^ calculated after exclusion of patients in the Unknown category.

### Lung and bronchial cancer incidence rates

**Fig 1:**
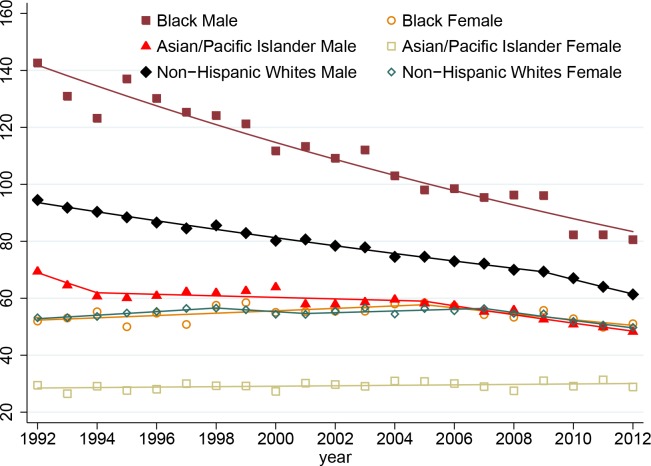
Age-adjusted lung and bronchial cancer incidence rates in the SEER 13 registries 1992–2012). The rates are presented per 100,000 individuals and age-adjusted according to the U.S. population in 2000 (19 age groups; Census P25-1130).

Fig 1 shows the age-adjusted lung and bronchial cancer incidence rates according to race and sex in patients in the SEER 13 registries (1992–2012). The incidence rates in male patients were markedly higher than those in female patients in the same ethnic groups. Overall, male patients had higher incidence rates than female patients did in most of the ethnic groups. The incidence rates in male patients in each group continuously decreased from 1992 to 2012, but the rates in female patients remained relatively stable during that period, even increasing slightly in black patients from 1992 to 2005. Although the greatest decrease in incidence rate occurred in male black patients, male and female black patients had the highest incidence rates in the sex subgroups, whereas male and female Asian patients had the lowest incidence rates in those subgroups.

### Survival

The median follow-up duration in the study cohort was 0.58 years (mean, 1.65 years; range, 0–22.90 years). The 5- and 10-year DSS and OS rates are shown in Table 3. Overall, Asian patients had the best 5-year DSS (24.7%) and 5-year OS (17.6%) rates, whereas black patients had the worst 5-year DSS (20.4%) and 5-year OS (13.2%) rates. HW patients had better 10-year DSS and OS rates (18.4% and 9.5%, respectively) than did NHW (17.9% and 8.7%, respectively) and black (14.9% and 6.9%, respectively) patients. We found statistically significant heterogeneity in the survival rates among the eight Asian subgroups and among the NHW, black, and HW groups. Indian/Pakistani patients had the best 5-year DSS rate (31.3%), whereas black and Hawaiian/Pacific Islander patients had the worst 5-year DSS rates (20.4% and 20.1%, respectively) followed by Japanese (22.6%) and Korean (21.9%) patients. All of the Asian subgroups except Hawaiian/Pacific Islander (13.9%) and Japanese (15.8%) patients had better 5-year OS rates than did the NHW, black, and HW patients (16.1%, 13.2%, and 16.0%, respectively).

**Table 3:** Five- and 10-year survival rates in the patients with lung or bronchial cancer by ethnic group.

	% (95% confidence interval)			
Ethnic group	5-year DSS rate	10-year DSS rate	5-year OS rate	10-year OS rate
**NHW**	23.7 (23.6–23.8)	17.9 (17.8–18.1)	16.1 (16.0–16.2)	8.7 (8.6–8.8)
**Black**	20.4 (20.1–20.8)	14.9 (14.6–15.3)	13.2 (12.9–13.4)	6.9 (6.7–7.2)
**HW**	23.6 (23.0–24.1)	18.4 (17.8–19.0)	16.0 (15.6–16.5)	9.5 (9.1–10.0)
**Asian**	24.7 (24.2–25.2)	18.1 (17.6–18.7)	17.6 (17.2–18.0)	10.4 (10.0–10.8)
Filipino	27.8 (26.6–28.9)	20.7 (19.4–22.0)	17.7 (16.8–18.6)	10.7 (9.9–11.5)
Japanese	22.6 (21.4–23.8)	17.0 (15.8–18.3)	15.8 (14.8–16.8)	9.1 (8.3–10.0)
Chinese	24.1 (23.1–25.2)	16.8 (15.7–18.0)	18.1 (17.3–19.0)	10.6 (9.8–11.5)
Hawaiian/Pacific Islander	20.1 (18.6–21.6)	15.5 (14.0–17.2)	13.9 (12.7–15.1)	7.6 (6.6–8.7)
Korean	21.9 (20.0–23.7)	14.9 (13.1–16.9)	17.0 (15.5–18.6)	9.7 (8.4–11.2)
Indian/Pakistani	31.3 (28.0–34.6)	27.5 (23.8–31.3)	23.8 (21.0–26.6)	18.5 (15.5–21.6)
Vietnamese	25.3 (23.5–27.2)	18.6 (16.7–20.6)	19.2 (17.7–20.8)	11.7 (10.3–13.3)
Other	28.5 (26.3–30.8)	20.7 (18.1–23.4)	21.8 (19.9–23.7)	13.0 (11.1–15.1)

We used multivariable Cox proportional models to conduct a stratification analysis of the patients’ clinicopathologic and ethnic variables contributing to DSS according to age (<50, 50–59, 60–69, and ≥70 years) and disease stage (I, II, III, and IV) (Tables 4 and 5) and found that race and tumor histology were factors associated with DSS. For example, patients with BAC histology had better DSS rates in each age group and at each disease stage than did patients with adenocarcinoma histology. Also, patients with squamous cell or large cell histology had worse DSS rates in each age group and at each disease stage than did patients with adenocarcinoma histology (data not shown). We observed the differences in DSS rate among the four broad ethnic groups (Table 4) and eight Asian subgroups (Table 5) in the different age and disease-stage groups, as well. Compared with NHW patients, black patients had worse DSS rates in almost every age and disease-stage group, especially in the younger groups (<50 and 50–59 years) and in the stage I group. Furthermore, compared with the other broad ethnic groups, Asian patients had better DSS rates in almost in every age and disease-stage group, especially in the older groups (50–59, 60–69, and ≥70 years) and at advanced stages (III and IV). We observed the DSS disadvantage in Japanese, Hawaiian/Pacific Islander, and Korean patients chiefly in the older age groups (60–69 and ≥70 years) and stage I, III, and IV groups. In addition, Hawaiian/Pacific Islander patients had the worst DSS rates in almost every age group and in the stage I, III, and IV groups. The DSS advantage in Indian/Pakistani patients was chiefly reflected in the younger age groups (<50 and 50–59 years) and in each disease-stage group.

**Table 4:** Multivariable analysis of clinicopathologic and ethnic variables associated with DSS using a Cox proportional hazards model for four age groups and four disease-stage groups.

	Age, years							
	50		50–59		60–69		≥70	
	Hazard Ratio	P	Hazard Ratio	P	Hazard Ratio	P	Hazard Ratio	P
	Stage I							
Ethnic group								
NHW	Referent		Referent		Referent		Referent	
Black	1.39	<0.0001	1.24	<0.0001	1.13	<0.0001	1.13	<0.0001
HW	0.72	0.03	0.75	0.001	0.97	0.56	0.98	0.65
Asian	0.86	0.27	0.83	0.008	0.77	<0.0001	0.90	<0.0001
	Stage II							
Ethnic group							Referent	
NHW	Referent		Referent		Referent			
Black	1.26	0.03	1.05	0.43	1.06	0.25	1.03	0.47
HW	0.54	0.04	0.98	0.85	0.94	0.50	0.98	0.80
Asian	1.19	0.32	0.95	0.63	0.91	0.21	0.88	0.03
	Stage III							
Ethnic group								
NHW	Referent		Referent		Referent		Referent	
Black	1.03	0.32	1.09	<0.0001	1.06	0.001	1.01	0.40
HW	0.88	0.03	1.02	0.61	0.99	0.62	1.01	0.82
Asian	0.89	0.04	0.89	<0.0001	0.88	<0.0001	0.90	<0.0001
	Stage IV							
Ethnic group								
NHW	Referent		Referent		Referent		Referent	
Black	1.05	0.01	1.03	0.03	1.00	0.96	0.98	0.04
HW	0.79	<0.0001	0.89	<0.0001	0.94	0.001	0.95	0.002
Asian	0.76	<0.0001	0.73	<0.0001	0.79	<0.0001	0.86	<0.0001

**Table 5:** Multivariable analysis of Asian subgroups associated with DSS using a Cox proportional hazards model for four age groups and four disease-stage groups.

	Age, years							
	50		50–59		60–69		≥70	
	Hazard Ratio	P	Hazard Ratio	P	Hazard Ratio	P	Hazard Ratio	P
	Stage I							
Ethnic group								
Filipino	Referent		Referent		Referent		Referent	
Japanese	1.02	0.97	0.50	0.02	1.10	0.52	0.98	0.79
Chinese	0.50	0.07	0.87	0.49	0.98	0.91	0.95	0.49
Hawaiian/Pacific Islander	0.85	0.69	1.21	0.36	1.83	<0.0001	1.34	0.01
Korean	0.59	0.39	0.60	0.15	1.72	0.002	1.08	0.52
Indian/Pakistani	-	-	0.60	0.28	1.01	0.98	0.75	0.17
Vietnamese	0.49	0.07	1.27	0.31	1.34	0.09	0.84	0.20
Other	0.55	0.28	1.08	0.79	0.96	0.87	0.82	0.15
	Stage II							
Ethnic group							Referent	
Filipino	Referent		Referent		Referent			
Japanese	1.06	0.93	0.94	0.88	0.87	0.51	1.16	0.38
Chinese	0.57	0.22	0.81	0.46	0.85	0.42	0.85	0.35
Hawaiian/Pacific Islander	0.59	0.49	0.86	0.64	0.76	0.30	0.67	0.18
Korean	0.82	0.73	1.58	0.26	0.64	0.08	1.06	0.83
Indian/Pakistani	-	-	0.52	0.37	1.60	0.32	1.04	0.91
Vietnamese	1.11	0.86	1.48	0.21	1.13	0.65	1.01	0.96
Other	0.89	0.86	0.74	0.44	0.69	0.33	1.49	0.15
	Stage III							
Ethnic group								
Filipino	Referent		Referent		Referent		Referent	
Japanese	0.93	0.73	1.01	0.93	1.16	0.047	1.32	<0.0001
Chinese	0.71	0.03	0.84	0.06	1.01	0.92	1.23	<0.0001
Hawaiian/Pacific Islander	1.38	0.03	1.11	0.32	1.41	<0.0001	1.28	0.001
Korean	0.85	0.45	1.13	0.31	1.25	0.02	1.23	0.004
Indian/Pakistani	0.84	0.51	0.79	0.22	0.81	0.18	1.09	0.48
Vietnamese	1.23	0.17	1.08	0.50	1.04	0.70	1.08	0.34
Other	0.98	0.91	1.23	0.13	1.02	0.88	1.02	0.85
	Stage IV							
Ethnic group								
Filipino	Referent		Referent		Referent		Referent	
Japanese	0.96	0.75	1.00	0.95	1.21	0.001	1.37	<0.0001
Chinese	0.76	0.002	0.82	0.002	0.99	0.82	1.13	0.002
Hawaiian/Pacific Islander	1.45	<0.0001	1.37	<0.0001	1.51	<0.0001	1.29	<0.0001
Korean	0.93	0.58	0.94	0.52	1.00	0.99	1.31	<0.0001
Indian/Pakistani	0.78	0.12	0.77	0.04	0.98	0.86	1.22	0.02
Vietnamese	1.06	0.55	0.93	0.37	1.00	0.99	1.03	0.60
Other	0.93	0.56	0.84	0.04	0.97	0.74	0.93	0.27

-: sample size too small

## Discussion

This study is one of the most comprehensive population-based analyses of lung and bronchial cancer according to ethnicity reported in the literature. With no precedent, we used data from the SEER database to examine a large cohort of patients with lung or bronchial cancer (including NSCLC and small cell lung cancer) throughout the United States over a 23-year period with a focus on four broad ethnic groups and eight heterogeneous subgroups of Asian patients to identify any differences in clinicopathologic features, treatment, and survival rates between Asian patients and patients in other ethnic groups. Another objective of our analysis was to determine whether any differences in survival could be attributed to disease-associated variables and, if so, identify the reasons for these differences. Consistent with previous studies [3–6,20], we showed that black patients had the poorest 5-year DSS and OS rates and higher rates of lymph node invasion, more advanced disease stages, fewer surgeries, more unfavorable histologic subtypes (squamous cell and large cell lung cancer), and less favorable histologic subtypes (adenocarcinoma and BAC) than did patients in the other ethnic groups. Researchers previously confirmed that most of these factors were predictors of poor survival in NSCLC patients [15,21–24]. Additionally, based on the results of stratification analysis of the patients’ clinicopathologic and ethnic variables (Table 4), we saw worse DSS rates in black patients than in patients in the other ethnic groups in nearly every age and disease-stage group.

We demonstrated that Asian patients had the best survival rates among the four broad ethnic groups, which was in line with previous reports [7–11,25,26]. The higher DSS rates in Asian patients than in the other three ethnic groups may be attributed to their favorable clinicopathologic features demonstrated in our analysis, including less advanced tumors, higher rates of surgical treatment, and more favorable histologic subtypes (adenocarcinoma and BAC). Investigators have confirmed that most of these factors are predictors of good survival in NSCLC patients [15,21–24]. Another conceivable reason for the good DSS results in Asian patients is a preponderance of epidermal growth factor receptor (EGFR) mutations in Asian lung cancer patients, especially East Asian NSCLC patients, than in non-Asian patients [27]. This factor also may be partially responsible for the finding that NHW patients had poorer survival rates than did Asian patients even though the former patients were more likely to be diagnosed with early-stage cancer and to undergo surgical treatment in our study. In a series of epidemiologic studies using information obtained from the California Cancer Registry database, Ignatius *et al* [10] consistently found that Asian ethnicity was an independent favorable prognostic factor for survival regardless of smoking status and treatment according to a Cox proportional hazards analysis of patients with early-stage NSCLC [28], advanced-stage NSCLC [26], or NSCLC at any stage [29]. Furthermore, in our stratification analysis of the patients’ clinicopathologic and ethnic variables (Table 4), we found that the DSS rate was consistently high in Asian patients in almost every age and disease-stage group. Moreover, the good DSS was more prominent in Asian patients in the older and advanced-stage groups who underwent few surgeries. One of the possible reasons is greater sensitivity of lung cancer to treatment with EGFR tyrosine kinase inhibitors in Asian patients than in non-Asian patients. This finding partially conflicted with the findings of a previous study with a much smaller sample size than ours from 2004 to 2010 that demonstrated that Asian NSCLC patients with early-stage (I and II) or stage IV disease had increased DSS rates [8].

The acculturation, socioeconomic status (SES), and cultural beliefs and practices regarding health care are highly diverse in the heterogeneous Asian population in the United States. Differences in these factors according to ethnicity may be largely responsible for well-documented ethnic disparities in disease survival [4,11,16,20,30]. Indeed, our findings demonstrated marked differences in clinicopathologic features, treatment, and survival rates among eight Asian subgroups of lung and bronchial cancer patients, suggesting that statistical analysis of survival in Asians as a single group is not informative in quantifying lung cancer burden and certainly not in guiding public health and clinical practice. In particular, we found that Indian/Pakistani patients had the best DSS rates because they had less advanced tumors than did patients in the other seven Asian subgroups, the most frequent use of surgical treatment, and more favorable histologic subtypes of cancer. The DSS advantage in Indian/Pakistani patients was most obvious in the younger age groups at each disease stage. Furthermore, Hawaiian/Pacific Islander patients had the worst DSS rates because they had the lowest rate of surgical treatment and more affected lymph nodes, more advanced tumors, and less favorable histologic subtypes of cancer than did patients in the other Asian subgroups We observed this DSS disadvantage in almost every age group and in the stage I, III, and IV groups. Another study demonstrated similar findings [11], with poorer survival of NSCLC in Korean, Japanese, and other Asian patients while better survival in South Asian, Chinese, Filipino, and Vietnamese patients. Looking at the patient-grouping method in that study, we found that South Asians consisted of patients defined as Indian/Pakistani patients in our study and that other Asians consisted of individuals we defined as Hawaiian/Pacific Islander patients. Thus, that study was generally consistent with ours.

Of note, we observed that among the East Asian patients in our study, Japanese and Korean patients had relatively poorer survival rates but higher surgery rates than did Chinese patients. This may be explained by unfavorable demographic and clinicopathologic features of the Japanese and Korean individuals in our analysis, including presentation with less favorable histologic subtypes of lung and bronchial cancer than those in the other groups. This result cannot be explained by lower SES or poorer health care access, as Japanese and Korean patients consistently had a higher SES and better access to health care than did the other Asian subgroups [11]. Thus, factors we could not assess, including important cultural differences and genetic variations that contribute to poor survival, may have been responsible for this finding. Another reason for it may be that Chinese patients were more likely than the other Asian patients to be diagnosed with adenocarcinoma or BAC, which are associated with more EGFR mutations than are the other histologic subtypes of lung cancer we studied. East Asian NSCLC patients have exhibited heterogeneous EGFR mutation rates of 24–40% for all histologic subtypes, with rates of 24.0% in Japanese patients [31], 26.7% in Korean patients [32], and 30.0% in Chinese patients [33].

NSCLC is a heterogeneous disease that is influenced by genetic, lifestyle, and socioeconomic elements. These elements are likely major factors affecting the disparate presentations and outcomes of the disease in different ethnic groups. The fact that lung cancer incidence is highest in male black individuals may be attributed to a variety of factors, including a higher smoking rate, more frequent single marital status, lower smoking quit rate, greater use of menthol cigarettes, lower SES, poorer access to quality health care, greater genetic susceptibility to tobacco carcinogens, and more common occupational and environmental exposure to lung carcinogens than in other ethnic groups [4]. All of these factors are also likely major contributors to the poor outcome of lung cancer in black and Hawaiian/Pacific Islander patients and the different lung cancer survival rates in different ethnic groups [8,11,20]. As some studies have demonstrated, with equivalent health care access, survival outcomes in different groups of lung cancer patients are comparable [20,34]. In addition, although lung cancer incidence rates in black and white women have been similar, the incidence rate in Asian women has been the lowest, which may be partially attributable to these women having the highest nonsmoking rate among all ethnic groups[35]. Since a marked reduction in smoking in male individuals in the 1960s [1], the lung cancer incidence rates in male members of all ethnic groups have decreased continuously as demonstrated in the present study.

Our study had some limitations. Because it was a retrospective investigation using a population-based database, we could not account for genetic or lifestyle factors linked with lung and bronchial cancer, including testing for mutations of EGFR, KRAS, ALK, and other driver genes; familial history of lung or bronchial cancer; smoking history; SES; and occupational exposure to carcinogens. This prevented us from evaluating these factors as potential confounders or effect modifiers in the observed relationships. In addition, age-adjusted lung cancer incidence rates in the SEER database were limited to the four broad ethnic groups because no data were available for the Asian subgroups. Also, data on cancer incidence rates were limited to the period from 1992 to 2012, which was when all data were available. Therefore, our analysis of data from 1990 and 1991 may have affected. Longer duration than that period may affect the results due to missing data and change of management. Although the SEER database covers 26% of black, 38% of Hispanic, 50% of Asian, and 67% of Hawaiian/Pacific Islander cancer patients, the findings for these ethnic groups in this study may not be generalized to the U.S. population, especially black and Hispanic members of it. Moreover, in view of ethnic assimilation into the SEER registry sites, the findings in the SEER database may minimize the actual differences in survival outcome.

In conclusion, our study demonstrated that the clinicopathologic features and treatment of lung and bronchial cancer differ by ethnicity in the United States and that the differences impact survival in each ethnic group.
